# Neuronal glycogen synthesis contributes to physiological aging

**DOI:** 10.1111/acel.12254

**Published:** 2014-07-25

**Authors:** Christopher Sinadinos, Jordi Valles-Ortega, Laura Boulan, Estel Solsona, Maria F Tevy, Mercedes Marquez, Jordi Duran, Carmen Lopez-Iglesias, Joaquim Calbó, Ester Blasco, Marti Pumarola, Marco Milán, Joan J Guinovart

**Affiliations:** 1 Institute for Research in Biomedicine (IRB Barcelona)Barcelona, Spain; 2 Department of Medicine and Animal Surgery, Autonomous University of BarcelonaBarcelona, Spain; 3 Center for Investigation in the Diabetes and Associated Metabolic Diseases Network (CIBERDEM)Barcelona, Spain; 4 Electron Cryo-Microscopy Unit, Scientific and Technological Centres, University of BarcelonaBarcelona, Spain; 5 Institució Catalana de Recerca i Estudis Avançats (ICREA)Barcelona, Spain; 6 Department of Biochemistry and Molecular Biology, University of BarcelonaBarcelona, Spain

**Keywords:** aging, *corpora amylacea*, *Drosophila*, glycogen, protein aggregation, stress response

## Abstract

Glycogen is a branched polymer of glucose and the carbohydrate energy store for animal cells. In the brain, it is essentially found in glial cells, although it is also present in minute amounts in neurons. In humans, loss-of-function mutations in laforin and malin, proteins involved in suppressing glycogen synthesis, induce the presence of high numbers of insoluble polyglucosan bodies in neuronal cells. Known as Lafora bodies (LBs), these deposits result in the aggressive neurodegeneration seen in Lafora’s disease. Polysaccharide-based aggregates, called *corpora amylacea* (CA), are also present in the neurons of aged human brains. Despite the similarity of CA to LBs, the mechanisms and functional consequences of CA formation are yet unknown. Here, we show that *wild-type* laboratory mice also accumulate glycogen-based aggregates in the brain as they age. These structures are immunopositive for an array of metabolic and stress-response proteins, some of which were previously shown to aggregate in correlation with age in the human brain and are also present in LBs. Remarkably, these structures and their associated protein aggregates are not present in the aged mouse brain upon genetic ablation of glycogen synthase. Similar genetic intervention in *Drosophila* prevents the accumulation of glycogen clusters in the neuronal processes of aged flies. Most interestingly, targeted reduction of *Drosophila* glycogen synthase in neurons improves neurological function with age and extends lifespan. These results demonstrate that neuronal glycogen accumulation contributes to physiological aging and may therefore constitute a key factor regulating age-related neurological decline in humans.

## Introduction

Glycogen, the storage polyglucosan (PG) in animal cells, is present in the brain at a fraction of the concentration found in muscle or liver (1/10 and 1/100, respectively, relative to these tissues) (Brown, 2004). So far, glycogen has been described primarily in astrocytes (Cataldo & Broadwell, 1986; Brown, 2004). However, neurons have also been recently shown to express both muscle glycogen synthase (MGS) and brain glycogen phosphorylase (BGP), key enzymes responsible for the production and degradation of glycogen (Saez *et al*., 2014). Neurons display an active glycogen metabolism and accumulate minute amounts of this polysaccharide (Saez *et al*., 2014).

Lafora’s disease (LD, *EPM2*) is a fatal neurodegenerative disorder caused by loss-of-function mutations in the laforin or malin proteins, which are involved in suppressing glycogen synthesis (Vilchez *et al*., 2007). In LD, high numbers of insoluble polyglucosan bodies (PGBs), known as Lafora bodies (LBs), are found in neurons (Lafora & Glueck, 1911). The extensive neuronal loss and severe neurological phenotypes observed in patients and animal models of LD (Delgado-Escueta, 2007; Valles-Ortega *et al*., 2011), suggest that neurons are particularly vulnerable to the excess of glycogen that accumulates in multiple tissues in this disease. Consistent with this proposal, neuron-specific hyperactivation of glycogen synthesis in cells and genetically modified mice and flies severely compromises neuronal function and survival (Vilchez *et al*., 2007; Duran *et al*., 2012).

The accumulation of PGBs, known as *corpora amylacea* (CA), has also been observed in the aged human brain (Cavanagh, 1999). CA share multiple histological and biochemical characteristics with LBs, including their composition of insoluble, poorly branched polysaccharide, resistance to digestion by amylase, and minor protein content [reviewed in (Cavanagh, 1999)]. Remarkably, the significance of PGB accumulation with regard to aging has been largely overlooked. In this regard, here, we address whether progressive PG accumulation in neurons during normal physiological aging is detrimental to neurological function and survival. We present evidence that the brains of aged mice accumulate glycogen-based aggregates that are similar to LBs in their localization and immunopositive response for an array of metabolic and stress-response proteins. Strikingly, these structures and their associated protein aggregates are abolished upon genetic ablation of MGS, the enzyme responsible for glycogen synthesis. Neuronal depletion of *Drosophila* glycogen synthase (dGS) also prevents the accumulation of glycogen clusters in the neuronal processes of aged flies, improves neurological function with age, and extends lifespan. Altogether, our observations reveal anabolic glycogen metabolism in neurons as a key contributor to physiological aging.

## Results

### MGS drives age-dependent PGB formation in the mouse brain

**Figure 1:**
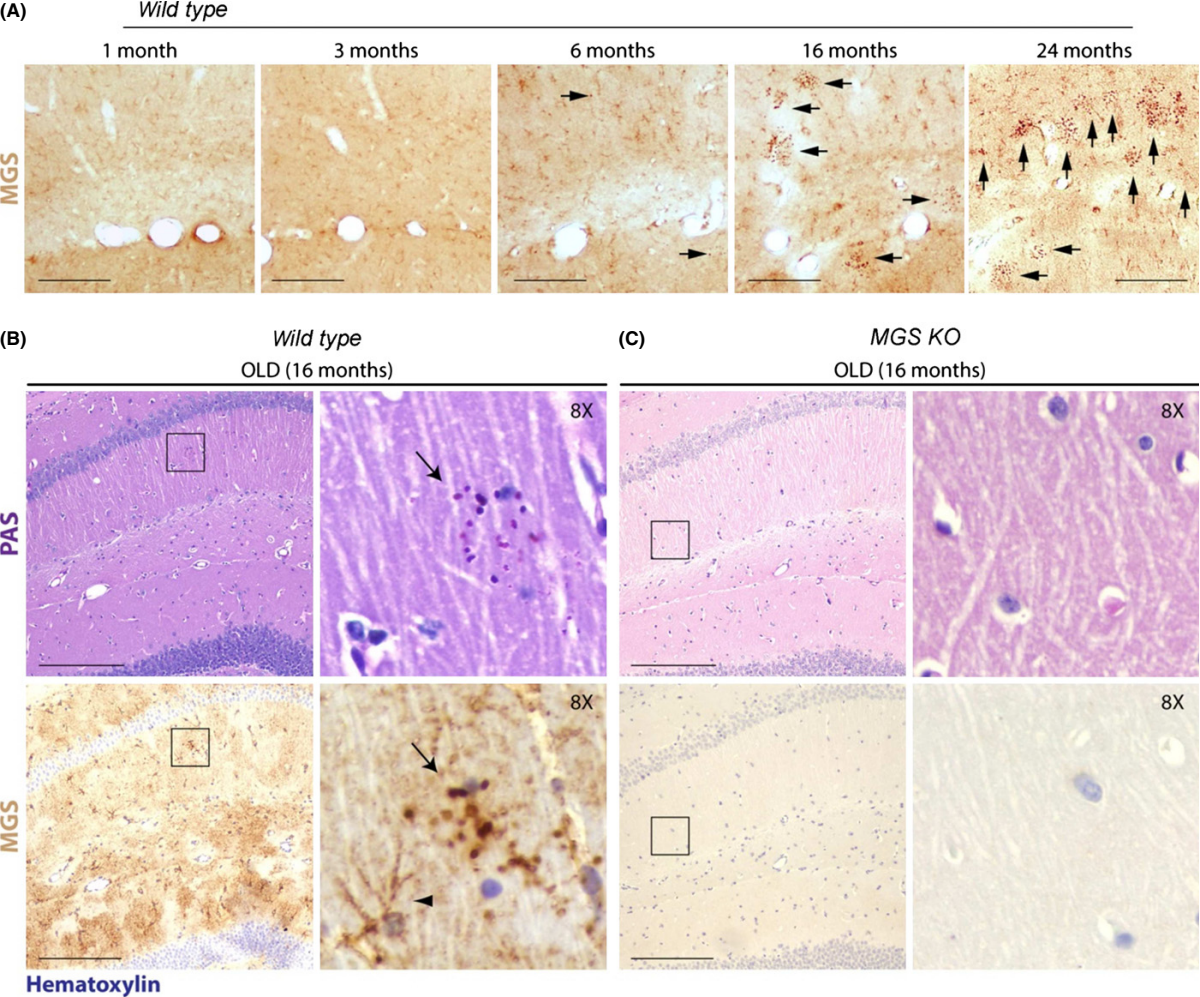
Age-dependent PG accumulation is GS-dependent. (A) 30-μm-thick (see Experimental procedures for details) brain (hippocampus) sections of 1-, 3-, 6-, 16- and 24-month-old *wild-type* mice (*n* = 3) were immunostained for MGS (brown). Scale bars = 100 μm. Arrows point to MGS accumulations. (B, C) Brain (hippocampus) sections of 16-month-old *wild-type* (A) and *MGS*^*KO*^ (B) mice. Examples of 4-μm-thick (see Experimental procedures for details) consecutive brain sections stained with periodic acid–Schiff (PAS) and immunostained for MGS (brown) as indicated (*n* = 3). All the sections showed in (B, C) were counterstained with hematoxylin (blue). Arrows point to PGBs, and arrowhead to an astrocyte showing normal MGS expression. Scale bars = 200 μm. Second and fourth row panels show magnifications of the squared fields.

CA-like structures in the brains of aged mammals are positive for the periodic acid–Schiff (PAS) stain, a marker of polysaccharides (Sakai *et al*., 1969a). These structures also show staining characteristics consistent with a minor protein component (Sakai *et al*., 1969a; Stam & Roukema, 1973; Suzuki *et al*., 1979a; Akiyama *et al*., 1986; King, 1994) and have collectively been termed polyglucosan bodies [PGBs, (Cavanagh, 1999)]. We analyzed the development of age-dependent PGBs in the brains of mice aged between 1 and 24 months and studied the contribution of glycogen metabolism to the accumulation of these deposits. The deposits, which were prevalent in aged mice, were positive for the PAS stain [Fig. 1B, (Akiyama *et al*., 1986)], a hallmark of human CA, and also immunopositive for MGS (Fig. 1A). As MGS is the only enzyme able to synthetize glucose polymers in mammals, we generated an MGS knockout mouse (*MGS*^*KO*^, see Fig. S1 (Supporting information) and Materials and Methods for details) and evaluated the relevance of MGS for the formation of these age-dependent PGBs in mouse brain. These mice registered a high perinatal mortality, an observation that is in agreement with findings for a similar model (Pederson *et al*., 2004). Nevertheless, surviving *MGS*^*KO*^ mice showed normal morphological development (data not shown). While PAS- and MGS-positive PGBs were found in the hippocampus, cerebellum, and piriform cortex of aged *wild-type* brains (Fig. 1B and Figs S2–S4), they were not found in any brain region of aged *MGS*^*KO*^ mice (Figs 1C and S2–S4). These findings indicate that MGS is responsible for the formation of age-dependent PAS-positive deposits in the mouse brain.

### Age-related PGBs in the mouse brain resemble human CA and LBs and accumulate stress-response proteins

**Figure 2:**
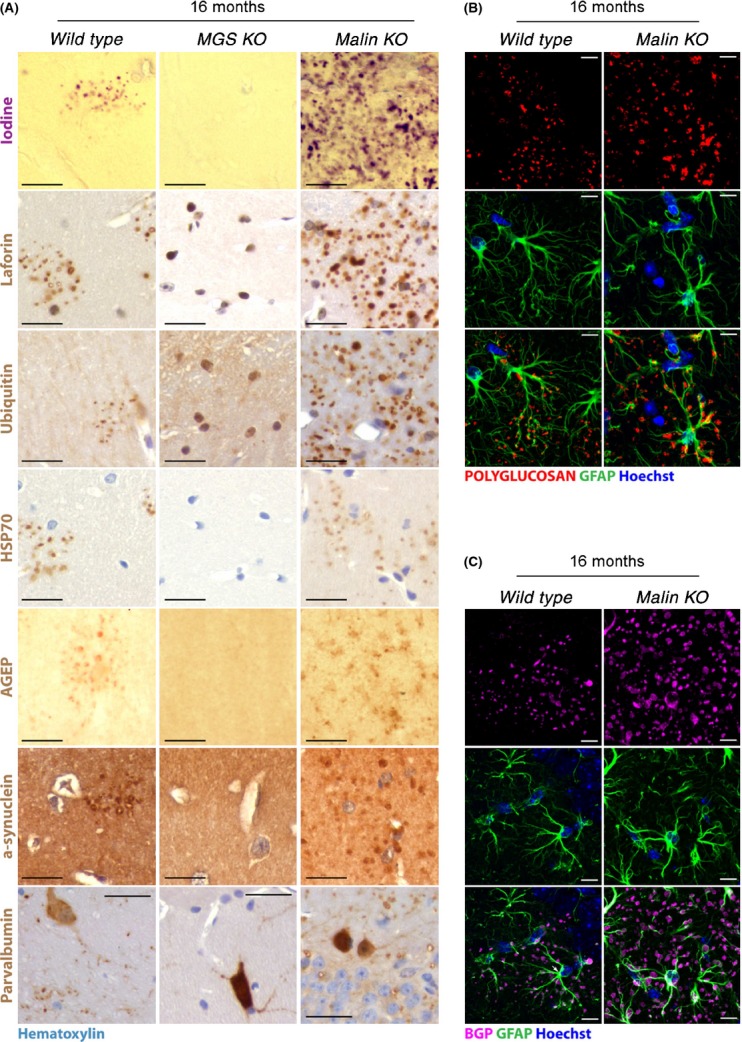
Consequences of MGS knockout on PGB accumulation during normal aging. (A) CA markers accumulated with polyglucosan bodies (PGBs) in aged (16-month-old) *wild-type* and *malin*^*KO*^ mice. Aged (16-month-old) *MGS*^*KO*^ mice did not show these accumulations. Hippocampus sections from *wild-type*, *malin*^*KO*^ and *MGS*^*KO*^ mice are shown (*n* = 3). 4-μm-thick sections (see Experimental procedures for details) consecutive to those stained with periodic acid–Schiff (PAS, not shown for clarity) were stained with iodine (purple) or immunostained with antibodies (brown) against laforin, ubiquitin, 70-kDa heat-shock protein (HSP70), advanced glycation end products (AGEP), alpha-synuclein, or parvalbumin. Immunostained sections were counterstained with hematoxylin (light blue). Scale bar = 25 μm. Laforin cellular localization appeared to be mainly nuclear in the absence of glucose polymers in *MGS*^*KO*^ brains. (B, C) PGBs in aged (16-month-old) *wild-type* and *malin*^*KO*^ mice accumulated glycogen phosphorylase (BGP) and were associated with astrocytes. Confocal images are shown for brains of 16-month-old *wild-type* and *malin*^*KO*^ mice. Antibodies were used against polyglucosan (red, B), brain glycogen phosphorylase (BGP, magenta, C), and glial fibrillary acidic protein (GFAP, green). Hoechst (blue) was used for nuclear staining. Scale bar = 10 μm. Hippocampus sections are shown in A–C.

The PGBs observed in the aged mouse brain were reminiscent of human CA in terms of their accumulation with age and PG-based composition. To further address the nature of these bodies, we analyzed *wild-type* and *MGS*^*KO*^ aged brains for multiple human CA markers and compared them with those of young *malin*^*KO*^ mice, an animal model of LD that rapidly accumulates large numbers of PGBs in the same brain areas as aged *wild-type* mice [(Valles-Ortega *et al*., 2011) and Figs S2–S4]. The deposits in aged *wild-type* and *malin*^*KO*^ brains, although larger and more abundant in the LD model, were equally positive for PAS staining (Figs S2–S4), iodine staining (Figs 2A and S2–S4) – which also stains PG (Reed *et al*., 1968; Sakai *et al*., 1969b) – and they immunoreacted with anti-PG antibody (Fig. 2B), thus confirming that they were PGBs. In addition to MGS (Fig. 1), other glycogen-binding proteins, including Laforin (Figs 2A and S2–S4) and BGP, the glycogen-degrading enzyme (Fig. 2C), accumulated on PGBs in the *wild-type* and *malin*^*KO*^ brains. Neither PG markers nor, as expected, MGS accumulated in any brain region of *MGS*^*KO*^ mice (Figs 2 and S2–S4).

Stress-related markers, such as ubiquitin (Cisse *et al*., 1993; Marquez *et al*., 2010), heat-shock proteins (Cisse *et al*., 1993; Iwaki *et al*., 1996; Marquez *et al*., 2010), and advanced glycation end products (AGEP) (Iwaki *et al*., 1996; Kimura *et al*., 1998), are present in human CA. Analysis of consecutively stained paraffin brain slices allowed us to conclude that the PGBs detected by PAS staining (not shown in Fig. 2A for clarity) in aged *wild-type* and *malin*^*KO*^ brains were also positive for ubiquitin, HSP70, and AGEP, while comparable accumulation of these markers was not detected in any region of aged *MGS*^*KO*^ mouse brains (Figs 2A and S5). It is unlikely that all cellular proteins indiscriminately associate with the PGBs in the aged mouse brain, as they were negative for several other markers (e.g. tau, NeuN, oligodendrocyte markers, and ß-amyloid, data not shown). The accumulation of alpha-synuclein, an aggregate-prone protein involved in Parkinson’s disease, has also been observed in PGBs (Trivedi *et al*. 2003; Uchida *et al*., 2003; Krim *et al*., 2005). In our hands, PGBs containing alpha-synuclein were found mainly in the hippocampus of aged *wild-type* brains and more ubiquitously in *malin*^*KO*^ brains (Figs 2A and S6B). Remarkably, no accumulation of alpha-synuclein was found in *MGS*^*KO*^ brains (Figs 2A and S6B).

Both glial and neuronal markers have been detected in human CA, leading to controversy as to the cell type of origin of these deposits (Cavanagh, 1999). We thus obtained confocal images from 16-month-old *wild-type* and *malin*^*KO*^ mice to compare PGB distribution among astrocytes and neuronal cells. Both types of mice showed abundant PGBs as aggregates reactive for antipolyglucosan (Fig. 2B) and anti-BGP (Fig. 2C) and surrounded by glial fibrillary acidic protein (GFAP)-positive processes. Parvalbumin (PV)-positive interneurons in the hippocampus of *wild-type* and *malin*^*KO*^ mice also accumulated PGBs (Figs 2A and S6B). Interestingly, no PV-containing aggregates were observed in *MGS*^*KO*^ mice, while the normal staining of interneurons was retained. This observation implies that PGBs that originate in these neurons during normal aging form in a GS-dependent manner. In summary, *wild-type* mice accumulate age-dependent PGBs in the brain that share multiple characteristics and staining properties with human CA and Lafora bodies and whose formation is MGS-dependent.

### GS contributes to PG accumulation in aged *Drosophila* brains

**Figure 3:**
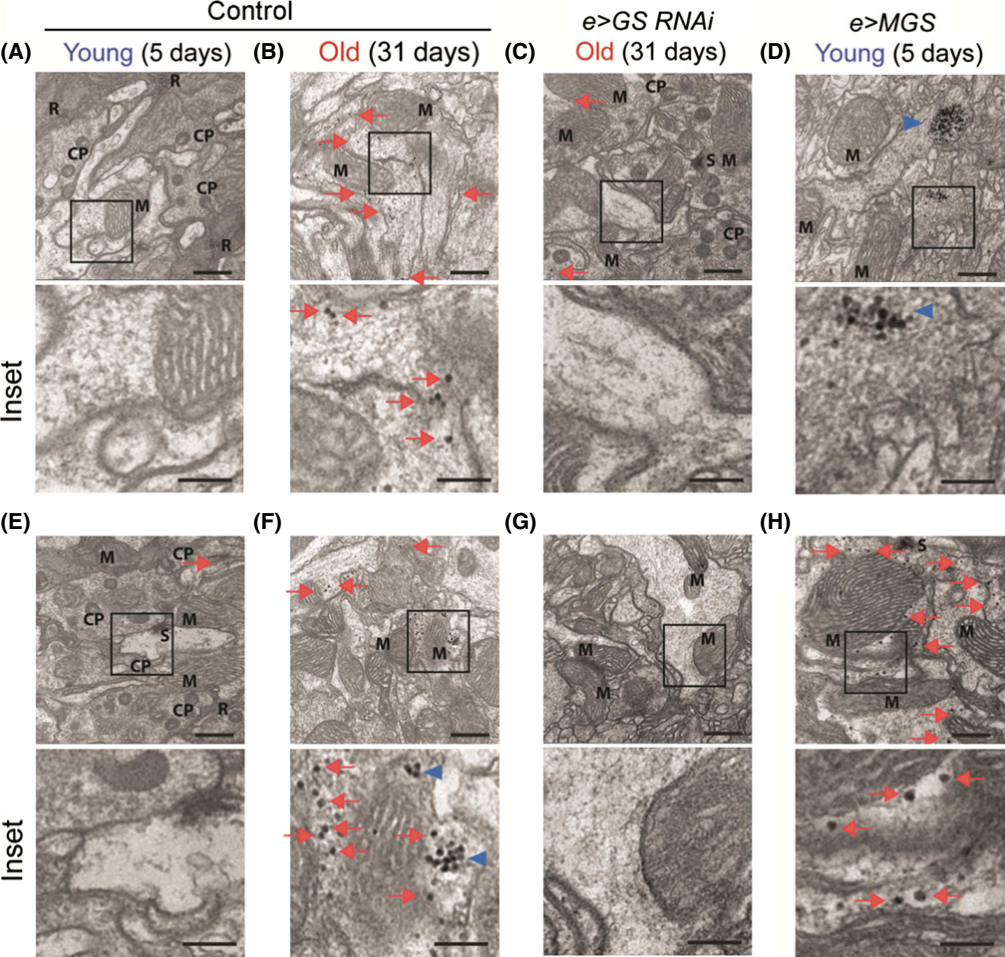
A role for GS in age-related PG accumulation in the *Drosophila* central nervous system.(A–H) Transmission electron microscopy (EM) analysis (×26 500) of optic lobe neuropils of young (5 days, A, D, E, H) and old (31 days, B, C, F, G) flies belonging to the following genotypes: *elav-Gal4/w*^*1118*^ (Control, A, B, E, F), *elav-Gal4/+; UAS-GS-RNAi-III/+* (e>GS-RNAi, C, G), and *elav-Gal4/+; UAS-MGS/+* (e>MGS, D, H). Red arrows point to glycogen granules and blue arrowheads to glycogen clusters. CP (capitate projection), M (mitochondrion near to glycogen), S (postsynaptic density), R (ribosomes in glial cell processes). Scale bar = 500 nm, inset 200 = nm. A–D, males, and E-H, females.

**Figure 4:**
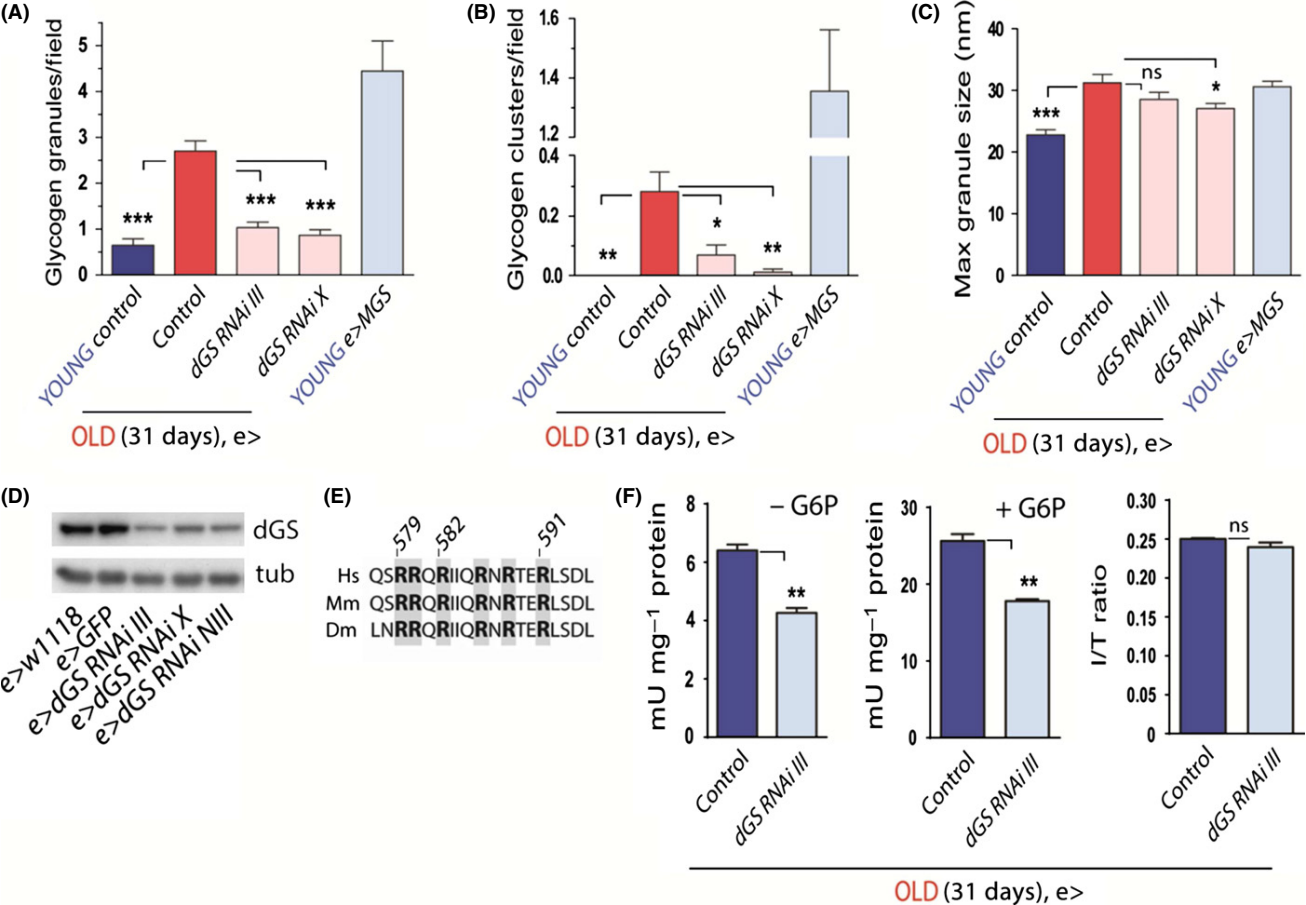
GS contributes to age-dependent PG accumulation in *Drosophila* brains. (A, B) Quantification of glycogen granules (A) and clusters (B) from TEM images. Young (5 days) and old (31 days) fly groups are shown in blue and red, respectively. (C) Quantification of maximum glycogen granule diameter as an average of the 10 largest granules identified for each group. Significance is versus aged (31d) group. In A–C, *n* = X,Y,Z where X = animals, Y = images, Z = neuronal processes. Control (young) *n* = 470 436; control (old) *n* = 5116 676; dGS-RNAi-III (old) *n* = 5144 1006; dGS-RNAi X (old) *n* = 390 730, MGS (young) *n* = 344 231. (D) Determination of dGS expression levels in young (5 days) dGS-RNAi animals by Western blot from whole head homogenates. Western blot membranes were exposed to anti-hMGS antibody (3886 clone, Cell Signaling, 1/1000). tub – tubulin loading control. Quantification in A–C is from pooled data of 2 males and 2 females (young, control), 2 male and 3 females (old, control), 4 females and 1 male (old, *dGS-RNAi-III*), 4 females (old, *dGS*-*RNAi X*), and 2 males and 1 female (young, *MGS*). (E) Alignment of amino acid sequences in human (Hs), mouse (Mm), and *Drosophila* (Dm) for comparison of the arginine-rich cluster involved in binding of the allosteric activator glucose-6-phosphate (G6P). Amino acid sequence numbers correspond to human MGS. Essential arginine residues for G6P activation are highlighted in gray. (F) GS activity ratio (−G6P/+G6P). Values were calculated from triplicate activity measurements in a single experiment (*n* = 60 fly heads, 30 each sex, per genotype). For all panels, e> denotes *elav-GAL4* pan-neuronal driver, ′control′ refers to *elav-Gal4 > w1118* flies, blue relates to young flies (5d old) and red to aged flies (31d old). Genotypes: *elav-Gal4/w*^*1118*^ (control), *elav-Gal4/+;* *UAS-dGS-RNAi-III/+* (e>dGS-RNAi-III), *elav-Gal4/UAS-GS-RNAi-X* (e>dGS RNAi X), *elav-Gal4/+; UAS-MGS* (e>MGS), and *elav-Gal4/+; UAS-dGS-RNAi-NIG-III/+* (e>dGS-RNAi-NIII), *elav-Gal4/+; UAS-GFP/+* (e>GFP),. *** *P* < 0.001, ** *P* < 0.01, * *P* < 0.05, ns – not significant, and data are expressed ±SEM.

**Figure 5:**
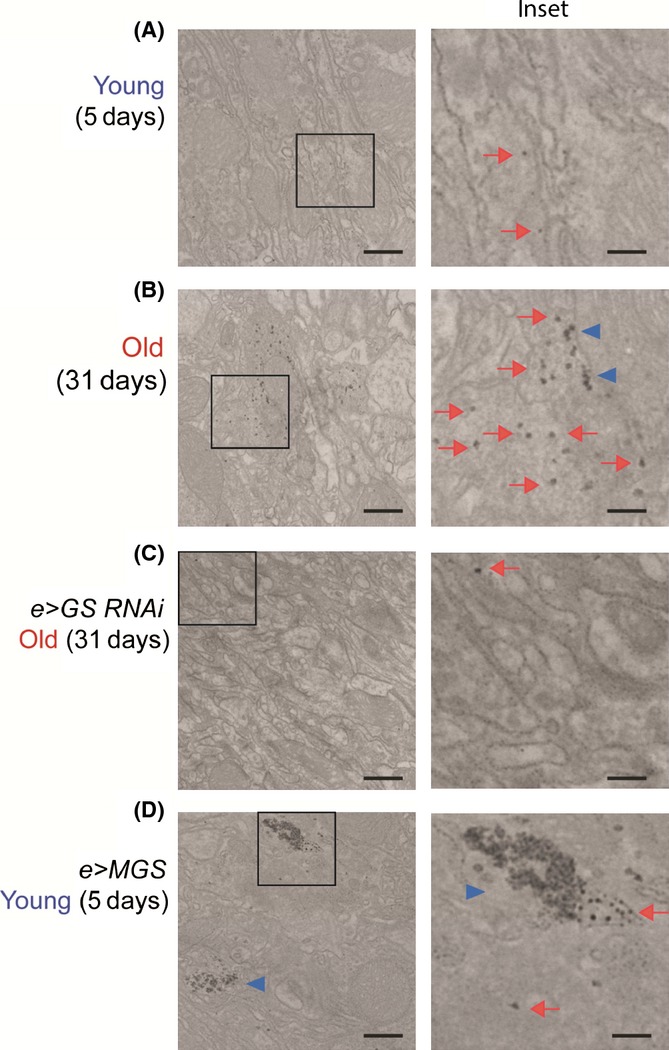
PAS-coupled silver staining contrasts glycogen granules in aged fly neurons. EM images (×15 000–×21 000) of optic lamina neuropil from ultrathin sections of male fly brains stained by the Thiéry method. Flies were young (5 days, A, D) or old (31 days, B, C) and belonged to the following genotypes: *elav-Gal4/w*^*1118*^ (Control, A, B), *elav-Gal4/+; UAS-dGS-RNAi-III/+* (*e>GS-RNAi*, C), and *elav-Gal4/+; UAS-MGS/+* (*e>MGS*, D). Scale bar = 500 nm, inset 200 = nm. Red arrows point to glycogen granules and blue arrowheads to glycogen clusters.

In order to establish a system in which to test the functional consequences of PG accumulation with age, we modeled the process in *Drosophila melanogaster*. *Drosophila* displays age-dependent cellular changes in the brain that are consistent with those observed in mammalian models and human nervous tissue, including the formation of membranous stacks and whorls, a reduction in the density of neuronal cytoplasm, and vacuole formation (Miquel *et al*., 1981). As flies are relatively short-lived in comparison with mice, we focused on neuronal glycogen granule distribution as a sensitive measure of early PG accumulation, particularly as these granules accumulate in aging mammalian axons where they locally associate with larger CA aggregates and may therefore precede CA formation (Suzuki *et al*., 1979b; King, 1994). We used electron microscopy (EM) to study the neuronal processes of the optic lobe neuropil, as this fibrous network is well-described anatomically (Edwards & Meinertzhagen, 2010) and contains neuronal processes like those that harbor CA in the human brain (Cavanagh, 1999). Glycogen granules were identified in this region on the basis of size (20–30 nm diameter), sphericity, and high density after postfixation in selected conditions, an established methodology for the detection of neuronal glycogen (Cataldo & Broadwell, 1986). Glycogen granules were rare in young fly brains (Fig. 3A,E) but readily identifiable in the neuronal processes of male and female aged flies (Fig. 3B,F, arrows). Clusters of glycogen granules were also observed in aged animals but not in young ones (Fig. 3A,B,E,F, arrow heads). The presence of isolated glycogen granules and clusters was significantly higher in aged flies than in young counterparts (quantified in Fig. 4A,B). The maximum glycogen particle size was also increased with age (Figs 4C and S7). The same type of granules, but more abundant, were observed in flies expressing human MGS in neurons [Fig. 3D,H, quantified in Fig. 4A,B, see also (Duran *et al*., 2012)]. To further test the hypothesis that the electron-dense particles identified in aged fly neurons were glycogen, we sequentially stained fly brain sections with periodic acid, thiosemicarbazide, and silver proteinate, following the method described by Thiéry (Rybicka, 1996), see Materials and Methods for details). This protocol couples PAS-reactive polysaccharides to dense silver–protein conjugates. Thiéry-positive granular structures were present in aged but not young fly neurons, as shown by EM (Fig. 5A,B, red arrows indicate glycogen granules and blue arrowheads glycogen clusters). This result is consistent with being glycogen. Thiéry-positive glycogen granules formed extensive accumulates in MGS-expressing animals (Fig. 5D), and granules of a similar size and shape were found in a positive control sample from mouse liver using this technique (Fig. S7). These observations collectively indicate that PG accumulation in neurons during aging is conserved in the fly.

*Drosophila* possesses a single homolog of human glycogen synthase, dGS (CG6904), which shows 78% similarity and 61% identity to the human MGS gene. To manipulate dGS levels specifically in neurons so as to test its effect on PG formation during normal physiological aging, we used the UAS-GAL4 system (Brand & Perrimon, 1993) and crossed three publically available *UAS-dGS* short hairpin RNAi lines (see Materials and Methods) with the pan-neuronal driver *elav-GAL4*. Using a monoclonal antibody raised against a fragment of human MGS that cross-reacts with the *Drosophila* protein (Fig. S8), we confirmed a 35–45% reduction in dGS protein levels in whole head homogenates of *elav-GAL4; UAS-dGS*-*RNAi* flies (*e>dGS-RNAi*, Figs 4D and S8). As MGS activity is highly regulated, we also assessed the activation state of the enzyme. The arginine cluster known to be essential for the allosteric activation of mouse MGS by G6P (Bouskila *et al*., 2010) was conserved in the fly (Fig. 4E). We measured the incorporation of C^14^-labeled glucose into glycogen by dGS from whole fly head homogenates. Both total dGS (+G6P, which allosterically activates all the GS present, thus allowing an estimation of total GS) and the level of endogenously active enzyme (-G6P) were reduced by approximately 35% in the heads of *e>dGS-RNAi* flies (Fig. 4F, *P* < 0.01, *n* = 3). As an estimated 10% of the cells in the *Drosophila* brain are glia (Edwards & Meinertzhagen, 2010) and the head of the fly also contains non-neural tissues likely to express dGS (Wigglesworth, 1949), this quantification of neuronal dGS knockdown is likely to be an underestimate. Furthermore, we did not detect any change in the proportion of active to total enzyme (-G6P/+G6P ratio) in *e>dGS-RNAi* fly heads (Fig. 4F), suggesting that there was no compensatory activation of the enzyme in response to dGS knockdown in these experiments.

Remarkably, isolated glycogen granules and clusters were reduced in the brains of aged *e>dGS-RNAi* flies versus the age-matched control genotype (compare Fig. 3B,F with C,G, see quantification in Fig. 4A,B). PG deposits were not the result of GAL4 or general transgene expression as they were also present in aged *w*^*1118*^ background strain animals (Fig. S9). Thiéry-positive glycogen granules were also relatively sparse in aged dGS-RNAi fly brain neurons when compared to age-matched controls (Fig. 5B,C). These observations together imply that *Drosophila* neurons accumulate PG in the brain with age and that dGS contributes to this phenomenon.

### Neuronal GS knockdown promotes healthy aging in *Drosophila*

**Figure 6:**
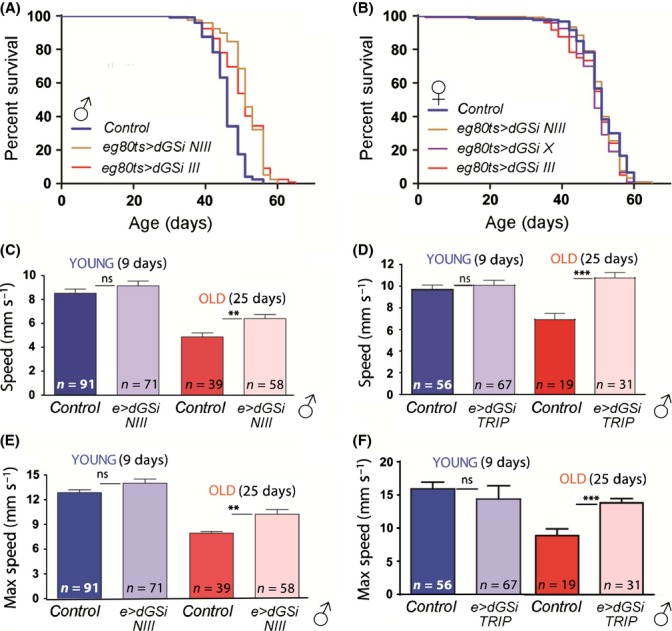
Functional consequences of reduced GS in the nervous system of aging *Drosophila*. (A, B) Kaplan–Meier survival curves of dGS RNAi lines driven with elav for male (A) and female (B) flies. Adult-specific RNAi was achieved by co-expressing gal80^ts^ and raising animals at 18 °C until adulthood. Genotypes: *elav-Gal4/w*^*1118*^ (control), *elav-Gal4/+;* *UAS-GS-RNAi-NIG-III/tub-gal80ts* (e g80ts>dGSi NIII), *elav-Gal4/+; UAS-GS-RNAi-III/ tub-gal80ts* (e g80ts>dGSi III), and *elav-Gal4/ UAS-GS-RNAi-X; tub-gal80ts/+* (e g80ts>dGSi X). Median lifespans and number of animals tested: (A) control 46 d, *n* = 120; e g80ts>dGSi III 51 d, *n* = 120; e g80ts>dGSi NIII 51 d, *n* = 120; (B) control 51 d, *n* = 97; e g80ts>dGSi III 51 d, *n* = 120; e g80ts>dGSi NIII 51 d, *n* = 120; e g80ts>dGSi X 49 d, *n* = 120. (C–F) Average (C, D) and maximum (E, F) climbing speed of young (9 d, blue) and old (25 d, red) male flies for *dGS RNAi NIII* (C, E) and *dGS RNAi TRiP* (D, F) lines driven with *elav-Gal4* in neurons. Genotypes: *elav-Gal4/+; UAS-GFP/+* (control, C, E), *elav-Gal4/+; UAS-GS-RNAi-NIG-III/+* (e>dGSi NIII, C, E), *elav-Gal4/+; UAS-GFPval/+* (control, D, F), *elav-Gal4/+; UAS-GS-RNAi*-TRiP/+ (e> dGSi TRIP, D, F). ** *P* < 0.01, *** *P* > 0.001.

We next assessed whether PG accumulation with age has relevant consequences for neurological function *in vivo*. We first analyzed the longevity of adult flies expressing *dGS-RNAi* in neurons. Three strains harboring dsRNA forms of dGS and whose lifespan profiles were similar to control flies (Fig. S10C,D) were backcrossed eight times into the *w*^*1118*^ genetic background. Backcrossed lines were crossed with the neuronal *elav-Gal4* driver, and the longevity of the progeny was measured. We used the Gal80ts TARGET system (McGuire *et al*., 2003) to drive conditional expression during adult life only (Fig. S10B). Adult males expressing *dGS-RNAi* displayed a significantly longer median (log-rank test) and maximum (Wang–Alison analysis) lifespan than driver-only expressing controls (Fig. 6A, 10% increase in median lifespan, *P* < 0.0001, see also Table S1). No significant increase in lifespan was detected in females expressing *dGS-RNAi* (Fig. 6B). To assess the neurological capacity of young and aged flies, we next analyzed adult climbing behavior. Young males expressing *dGS-RNAi* displayed a normal climbing response, with no significant change in climbing speed relative to GFP-expressing control flies (Fig. 6C). By contrast, aged males showed a higher average and maximum climbing speed than control GFP-expressing flies (*e>GFP,* Fig. 6C and Movies S1–S4). This difference was not due to deleterious effects of neuronal GFP expression with time in *e>GFP* control flies, as it was also observed when *dGS-RNAi* expressing flies were compared to *w*^*1118*^ heterozygous or *lacZ*-expressing control strains (Fig. S10). A *dGS-RNAi* TRiP line, which is on a distinct genetic background to the above RNAi lines, also displayed an improved climbing performance in aged animals versus controls when driven by *elav-Gal4* (Fig. 6D). The increase in average climbing velocity was also reflected by a higher maximum climbing velocity for aged *dGS-RNAi* expressing flies (Fig. 6E,F). No significant difference in the climbing performance was observed in aged *dGS-RNAi* expressing female flies when compared to controls (data not shown). Taken together, these findings indicate that dGS function in neurons impairs neurological function and survival with age.

## Discussion

Here, we have established a positive correlation between neuronal glycogen synthesis and aspects of the pace of physiological aging in invertebrate and mammalian animal models. Furthermore, we reveal that age-related PG accumulation resembles the first stages of the rapid, massive deposition of glycogen observed in animal models of Lafora disease. In this rare and lethal hereditary condition, the accumulation of abnormal glycogen underlies the neurodegeneration characteristic of this disease (Duran *et al*., 2014). However, such an alteration has not been contemplated in the context of glycogen metabolism in neurons of the normal human brain. In this regard and given that CA in the aged human brain occurs in neurons as well as in glial cells (Woodford & Tso, 1980; Palmucci *et al*., 1982; Cavanagh, 1999), we analyzed the contribution of glycogen synthesis to CA formation and its consequences over time. Our results show that glycogen synthesis is a prerequisite for the formation of age-dependent PGBs in the mouse brain. As age-dependent PGBs in humans and other mammals are intimately associated with densely concentrated glycogen granules (Suzuki *et al*., 1979b; Woodford & Tso, 1980; Gertz *et al*., 1985; King, 1994), the accumulation of glycogen into clusters may represent a step in the formation of insoluble PGBs from glycogen. Several chemical characteristics of PGBs, including their insolubility and increased resistance to degradation by catabolic enzymes (Cavanagh, 1999), suggest that these aggregates persist in postmitotic neurons for long periods. This situation is reminiscent of the protein-based aggregates that accumulate in the brain in neurodegenerative diseases involving perturbed proteostasis and that also develop at a reduced pace during normal aging (David *et al*., 2010; Hartl *et al*., 2011). An important, and completely unexpected, finding of our work is that glycogen synthesis is causally implicated in the formation of protein-based aggregates, as age-dependent accumulations of several aggregation-prone or stress-response proteins, including alpha-synuclein, hsp70, and ubiquitin, were absent in *MGS*^*KO*^ mouse brain. The increased accumulation of these markers in the brains of young *malin*^*KO*^ mice, a model of LD, is consistent with this proposal. Collectively, our findings lead us to propose that progressive accumulation of glycogen in neurons is a widespread phenomenon in the aging human population that contributes to neurological decline and that LD resulting from rare mutations in malin and laforin drastically increases the rate of this process.

Accordingly, interventions that reduce the steady accumulation of neuronal glycogen would have a beneficial effect by slowing functional, age-related decline, as we have shown here in *Drosophila*. *Drosophila* also progressively accumulates PG in neuronal processes during its rapid aging. Specifically, aged flies, in a similar manner to mice, exhibit clusters of glycogen granules in neuronal processes that resemble those encountered in young flies with genetically enhanced PG synthesis (Duran *et al*., 2012). Neuron-specific knockdown of dGS reduced PG accumulation, improved locomotor capacity with age, and increased lifespan. The effect of dGS knockdown on longevity presented a marked sexual dimorphism, with substantial effects visible only in males. While the basis for this sex difference is not understood, sexually dimorphic genetic effects on *Drosophila* lifespan have been previously reported. Thus, increased Keap1/Nrf2 signaling, which regulates tolerance to oxidative stress, extends the lifespan of males but not females (Sykiotis & Bohmann, 2008). By contrast, females mutant in *chico*, the *Drosophila* homolog of vertebrate insulin receptor substrate (IRS), are long-lived, whereas mutant males are short-lived (Clancy *et al*., 2001). Our identification of glycogen granules in neuronal processes of male and female optic lobes leads us to propose that neuronal glycogen accumulation with age is a phenomenon common to both sexes, whereas the age-related response to this accumulation may be influenced by gender.

Our findings reveal a direct connection between neuronal glycogen synthesis and age-related functional decline in a multicellular organism. Altogether, our data indicate that glycogen accumulation in neurons during aging is an evolutionarily conserved process from flies to mammals. They also identify neuronal PG accumulation as a factor contributing to neuronal decline during aging in *Drosophila* and point to glycogen synthesis as a promising target to tackle the age-related deterioration of the nervous system.

## Experimental procedures

### Transgenic mouse generation

*Epm2b*-disrupted (*malin*^*KO*^) mice were generated as described previously (Valles-Ortega *et al*., 2011). *Gys1*-disrupted (*MGS*^*KO*^) ES C57BL/6N cells were obtained from the European Conditional Mouse Mutagenesis Program (EUCOMM), the Wellcome Trust Sanger Institute, Hinxton (UK). In these cells, the *Gys1* gene is disrupted by the insertion of a cassette containing the LacZ and NeoR genes between exons five and six (Fig. S1). After confirmation of targeting by PCR analyses, the cells were injected into C57BL/6J blastocysts, and these were then implanted in the uterus of pseudo-pregnant C57BL/6J females for the generation of chimeric mice. One chimeric male positive for the disruption was mated with C57BL/6J females to test for germline transmission. Heterozygous F1 mice were intercrossed to generate the animals used in this study. *Wild-type*, heterozygous, and homozygous null mice were identified by PCR genotyping.

### Mouse manipulation

All procedures were approved by the Barcelona Science Park’s Animal Experimentation Committee and were carried out in accordance with the European Community Council Directive and National guidelines for the care and use of laboratory animals. Mice were allowed free access to a standard chow diet and water and maintained on a 12-h/12-h light/dark cycle under specific pathogen-free conditions in the Animal Research Center at the Barcelona Science Park. Mice were weaned at 3 weeks of age, and tail clippings were taken for genotyping by PCR.

### Histology

Mice were anesthetized and perfused transcardiacally with phosphate-buffered saline (PBS) containing 4% paraformaldehyde (PF). Brains were removed and postfixed for 12 h with PBS–4% PF, embedded in paraffin and sectioned coronally at 4 μm. Four sections of each brain area under study were used per animal for staining or immunohistochemistry assays. To obtain cryosections, brains were cryoprotected after postfixation with PBS-30% sucrose, frozen, sectioned coronally at 30 μm, distributed in 10 series of 40–50 sections (representative of all the brain regions under study), and maintained at −20 °C in PBS–30% glycerol–30% ethylene glycol. A whole series of sections were used per animal for free-floating assays. Mouse brain sections were stained with hematoxylin and eosin (HE), periodic acid–Schiff (PAS) and Iodine (Lugol).

### Immunohistochemistry (IHC)

In IHC studies, the primary antibodies used were against MGS (1:200, Epitomics 1741), laforin (1:150, a generous gift from Dr. Rodríguez de Córdoba), advanced glycation end products (AGEP, 1:500, a generous gift from Dr. Rafael Salto), ubiquitin (1:300, Dakocytomation Z0458), HSP70 (1:50, MBL International Corporation SR810F), parvalbumin (PV, 1:3000, Sigma P3088), and α-synuclein (1:300, Chemicon AB5334P). To minimize the background, the MOM kit (Vector laboratories Inc BMK-2202) was used in monoclonal mouse primary antibody IHC. For IHC based on rabbit primary antibodies, the anti-rabbit Envision-System-HRP (Dakocytomation K4011) kit was used. In all cases, positive immunoreactivity was detected with the 3, 3’-diaminobenzidine tetrahydrochloride (DAB) system included in the Envision-System-HRP (Dakocytomation K4011) kit. All stainings were specific to the primary antibody.

### Immunohistofluorescence

Fluorescent immunodetection of antigens was performed by free-floating on 30-μm mouse brain sections that were washed in PBS and PBS–0.1% Triton X-100, blocked for 2 h at RT with PBS containing 10% of normal goat serum (NGS), 0.2% of gelatin, and F(ab’)_2_ fragment anti-mouse IgG when required. Primary antibodies were incubated overnight at 4 °C with PBS–5% NGS. We used antibodies against glial fibrillary acidic protein (GFAP, 1:500, Millipore MAB360), polyglucosan (1:50, Kamiya MC-253), and brain glycogen phosphorylase (BGP, 1:1000) (Vilchez *et al*., 2007). Dye-labeled secondary antibodies and Hoechst 33342 were incubated for 2 h at RT in PBS–5% NGS, mounted in Mowiol, and stored at −20 °C. Confocal images were taken with a Leica SP5 microscope and analyzed in imagej/fiji software. Three-dimensional representations were obtained by imaris® software (Bitplane AG, Zurich, Switzerland).

### Fly stocks

The *w*^*1118*^ (5905), *UAS-GFP* (4776), and *elav-GAL4* (8760) lines were from the *Drosophila* Bloomington Stock Center. UAS-dGS RNAi lines were from the Vienna *Drosophila* RNAi Center (III, v35136 and X, v35137), the National Institute of Genetics Japan (NIII, 6904R-3), or Bloomington Stock Center (TRiP, 34930). *elav-Gal4, tubulin-gal80*^*ts*^ flies were a kind gift from Dr. E. Skoulakis, Alexander Fleming Institute, Athens, Greece.

### Electron microscopy (EM) and protein analyses

For EM, adult flies were dissected live in 2% PFA with 2.5% glutaraldehyde fixative. Isolated brains were fixed in the same solution overnight at 4 °C and then postfixed in 1% osmium tetroxide supplemented with 0.8% potassium ferrocyanide for 2 h at 4 °C, conditions ideal for the contrasting of glycogen in neurons (Cataldo & Broadwell, 1986). Samples were dehydrated in ethanol series and embedded in epon resin. Ultrathin (50 nm) sectioning, poststaining, and imaging were as previously described (Duran *et al*., 2012). The neuropil of optic lobe lamina was identified on the basis of the presence of capitate junctions and/or the close proximity to retina. In this region, each neuronal process (area of membrane-delimited cytoplasm, relating to the profile of an axon or dendrite) was distinguished on the basis of a lighter electron density than surrounding glial projections. Glycogen granules were identified following a previous description of neuronal glycogen (Cataldo & Broadwell, 1986). Glycogen clusters were defined as instances where three or more glycogen granules were present in close proximity (<100 nm). Glycogen particle size was measured using the open source imagej/fiji software (Wayne Rasband, NIH, USA, version 1.47). For Thiéry staining, ultrathin sections prepared as above [or, in the case of mouse liver, as in (Fernandez-Novell *et al*., 1997)] were incubated 2 × 15 min in 10% periodic acid (PA), washed 5 × 5 min in mQ H_2_O, incubated overnight in 0.2% thiocarbohrydrazide in 20% acetic acid, washed 2 × 15 min in 10% acetic acid, 2 × 5 min in 5% acetic acid, 2 × 5 min in 1% acetic acid, 3 × 10 min in mQ H_2_O, and incubated for 30 min in 0.05 g silver proteinate in 5 mL mQ H_2_O, prior to final drop-wise washing of grids with mQ H_2_O. Transmission electron microscopy images were taken at 120 kv with a Tecnai Spirit electron microscope (FEI Company, Eindhoven, the Netherlands) equipped with a Megaview III CCD camera.

For Western blotting, protein levels were probed by standard procedures using anti-human GS 1/1000 (3886, Cell Signalling), anti-α tubulin 1/500 000 (DM1A Sigma-Aldrich Chemie GmbH, Buchs SG, Switzerland), and anti-GFP 1/600 (Molecular Probes). For comparison of human MGS, mouse MGS and *Drosophila* GS putative G-6-P binding regions, primary amino acid sequences were aligned in the ClustalW program using vector nti software (Invitrogen, Carlsbad, CA, USA). dGS activity was assessed from 60 adult heads per sample homogenized on ice in fresh GS activity buffer (Garcia-Rocha *et al*., 2001). Supernatant from this homogenate was assessed for C^14^ incorporation into glycogen over 30 min at 30 °C, as previously described (Valles-Ortega *et al*., 2011), using a specific activity of 1000 cpm μL^−1^ and 3000 cmp μL^−1^ for the measurement of T and I values, respectively.

### Climbing behavior

The climbing ability of flies was tested in an empty, 200-mm long, 25-mm wide clear cylindrical plastic tube at 25 °C and 60% humidity. Flies were tapped down and video-recorded using a CCD ORCA R2 camera (Hamamatsu, Japan) for up to 10 s with exposure time of 60 ms at 28.4 frames s^−1^ (binning two). Fly movies were exported as multipage TIF files and processed in imagej/fiji. Mean velocity was quantified posttesting using image background subtraction, contrast inversion, and particle tracker analysis features within a custom-made macro (Fly Tracker 1.0, developed by Sebastian Tosi, IRB Barcelona) in imagej/fiji. Average maximum climbing speed was assessed by selecting the 10 fastest flies from each sample group.

Gentoypes of flies used in these assays:

e>GFP w[*]/w[1118]; P{w[+mC]=UAS-GFP.nls}8/+; P{w[+mC]=GAL4-elav.L}3/+

e>dGS-RNAi-NIII w[*]/w[1118];; P{w[+mC]=GAL4-elav.L}3/ UAS-dGS RNAi-NIII

e>GFPval w[*]/y^*1*^ sc^*^ *v*^*1*^*; P{w[+mC]=GAL4-elav.L}3/P{UAS-GFP.VALIUM10}attP2*

e>dGS-RNAi TRiP w[*]/y[1] sc[*] v[1]; P{w[+mC]=GAL4-elav.L}3/P{y[+t7.7] v[+t1.8]=TRiP.HMS01279}attP2

### Lifespan analysis

Following the TARGET system (McGuire *et al*., 2003), three independent UAS-dGS-RNAi lines (UAS-dGS-RNAi-III, v35136, UAS-dGS-RNAi-NIG-III, 6904R-3, and UAS-dGS-RNAi-X, v35137), backcrossed eight times into the *w*^*1118*^ genetic background, were crossed with *elav-Gal4, tubulin-gal80*^*ts*^ flies, and progeny was developed at 18 °C. Newly eclosed adult flies were collected and kept in sex-specific tubes (max. 20 flies per vial, 120 flies per genotype) at 29 °C (to maximize RNAi hairpin expression during adulthood), 60% humidity and natural light/dark cycle conditions. To measure lifespans, deaths were scored upon tube changes every 2–3 d, before Kaplan–Meier lifespan curves were assembled and log-rank statistical analyses carried out. For individual comparisons, significance was assessed using isolated data from lifespan curves of interest with Bonferroni’s post-test statistical threshold corrections. Maximum lifespan was assessed using the Wang–Allison method, whereby a 2-way contingency table was generated and the significance (Fisher′s exact test) for living past the 90th percentile lifespan of all animals included in the analysis was assessed. All of the above statistical analyses were conducted with graphpad prism 4.0 software (GraphPad Software, Inc., La Jolla, CA, USA).

Genotypes of flies used in this analysis:

e g80ts>w1118 w[*], elav-Gal4/ w[1118]; +/+; tub-gal80ts/+

e g80ts>dGS-RNAi III w[*], elav-Gal4/w[1118];; tub-gal80ts/UAS-dGS-RNAi-III

e g80ts>dGS-RNAi-NIII w[*], elav-Gal4/ w[1118];; tub-gal80ts/ UAS-dGS RNAi-NIII

e g80ts>dGS-RNAi-X w[*], elav-Gal4/ w[1118] UAS-dGS-RNAi-X;; tub-gal80ts/+

## Abbreviations

AGEP: advanced glycation end product
CA: *corpora amylacea*
G6P: glucose-6-phosphate
GS: glycogen synthase
HSP: heat-shock protein
LD: Lafora’s disease
MGS: muscle glycogen synthase
PAS: periodic acid–Schiff
PG: polyglucosan
PGB: polyglucosan body
WT: wild-type

## Supporting Information

**Fig. S1** Generation of the MGS KO mouse. **Fig. S2** MGS and laforin proteins accumulated with polyglucosan bodies (PGBs) in the hippocampus of aged WT and malin KO mice. **Fig. S3** MGS and laforin proteins accumulated with polyglucosan bodies (PGBs) in the cerebellum of aged WT and malin KO mice. **Fig. S4** MGS and laforin proteins accumulated with polyglucosan bodies (PGBs) in the piriform cortex of aged WT and malin KO mice. **Fig. S5** Ubiquitin and 70 kDa heat-shock protein (HSP70) accumulated with polyglucosan bodies (PGBs) in aged WT and malin KO mice. **Fig. S6** Parvalbumin (PV) and alpha-synuclein accumulated with polyglucosan bodies (PGBs) in aged WT and malin KO mice. **Fig. S7** Glycogen accumulation in aged *Drosophila* as compared to mouse liver glycogen. **Fig. S8** Modulation of neuronal dGS levels detected in whole fly heads using anti-human MGS antibody. **Fig. S9** Identification of neuronal glycogen and glycogen clusters in aged *w*^*1118*^ strain flies. **Fig. S10** Data supporting neuronal RNAi knockdown of dGS and its functional consequences. **Table S1** Descriptive statistics for *Drosophila* lifespans

**Movie S1** Climbing of *elav-Gal4 > GFP* young flies.

**Movie S2** Climbing of *elav-Gal4 > dGS-RNAi-NIII* young flies.

**Movie S3** Climbing of *elav-Gal4 > GFP* aged flies.

**Movie S4** Climbing of *elav-Gal4 > dGS-RNAi-NIII* aged flies.
