# Supplementary material for: Neuronal glycogen synthesis contributes to physiological aging
Source: Aging Cell. 2014 Jul 25;13(5):935–45. doi: 10.1111/acel.12254 (PMC4331761; doi:10.1111/acel.12254)
Supplement: Supplementary file 1 — Fig. S1 Generation of the MGS KO mouse. Fig. S2 MGS and laforin proteins accumulated with polyglucosan bodies (PGBs) in the hippocampus of aged WT and malin KO mice. Fig. S3 MGS and laforin proteins accumulated with polyglucosan bodies (PGBs) in the cerebellum of aged WT and malin KO mice. Fig. S4 MGS and laforin proteins accumulated with polyglucosan bodies (PGBs) in the piriform cortex of aged WT and malin KO mice. Fig. S5 Ubiquitin and 70 kDa heat-shock protein (HSP70) accumulated with polyglucosan bodies (PGBs) in aged WT and malin KO mice. Fig. S6 Parvalbumin (PV) and alpha-synuclein accumulated with polyglucosan bodies (PGBs) in aged WT and malin KO mice. Fig. S7 Glycogen accumulation in aged Drosophila as compared to mouse liver glycogen. Fig. S8 Modulation of neuronal dGS levels detected in whole fly heads using anti-human MGS antibody. Fig. S9 Identification of neuronal glycogen and glycogen clusters in aged w1118 strain flies. Fig. S10 Data supporting neuronal RNAi knockdown of dGS and its functional consequences. Table S1 Descriptive statistics for Drosophila lifespans [file acel0013-0935-sd1.pdf]

Supporting Information

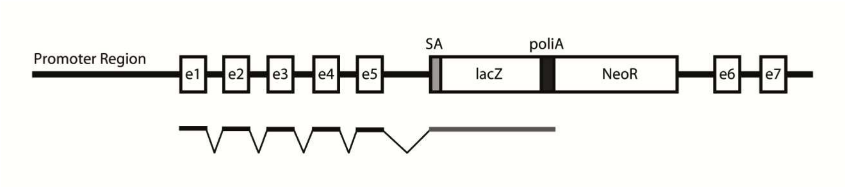

Figure S1. Generation of the MGS KO mouse. Schematic representation of the targeted disruption of *gys1* gene in MGS KO mice. The insertion of a selection cassette between exons 5 and 6 disrupts expression of the gene. See supplementary methods.

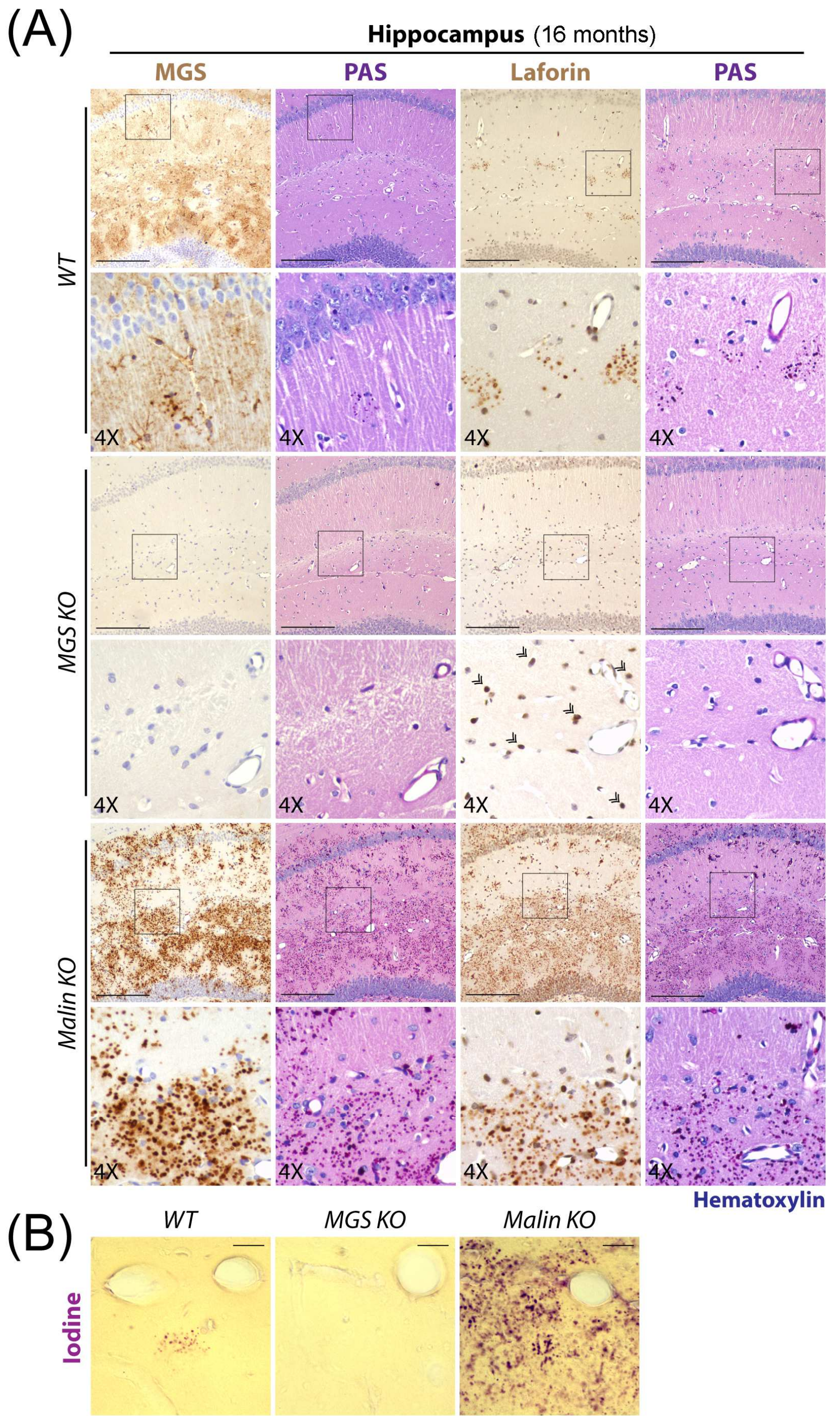

Figure S2. MGS and laforin proteins accumulated with polyglucosan bodies (PGBs) in the hippocampus of aged WT and malin KO mice. Aged MGS KO mice did not show accumulations. Hippocampi from16-month-old WT, malin KO and MGS KO mice are shown. (A) 4-µm-thick consecutive brain sections stained with periodic acid-Schiff (PAS) and immunostained for MGS and laforin (brown) as indicated. All the sections were counterstained

with hematoxylin (blue). Laforin staining presented nuclear localization in MGS KO mice (arrowheads). Scale bar = 200µm, 4X = 4-fold magnification. (B) Iodine staining. Scale bar = 30µm.

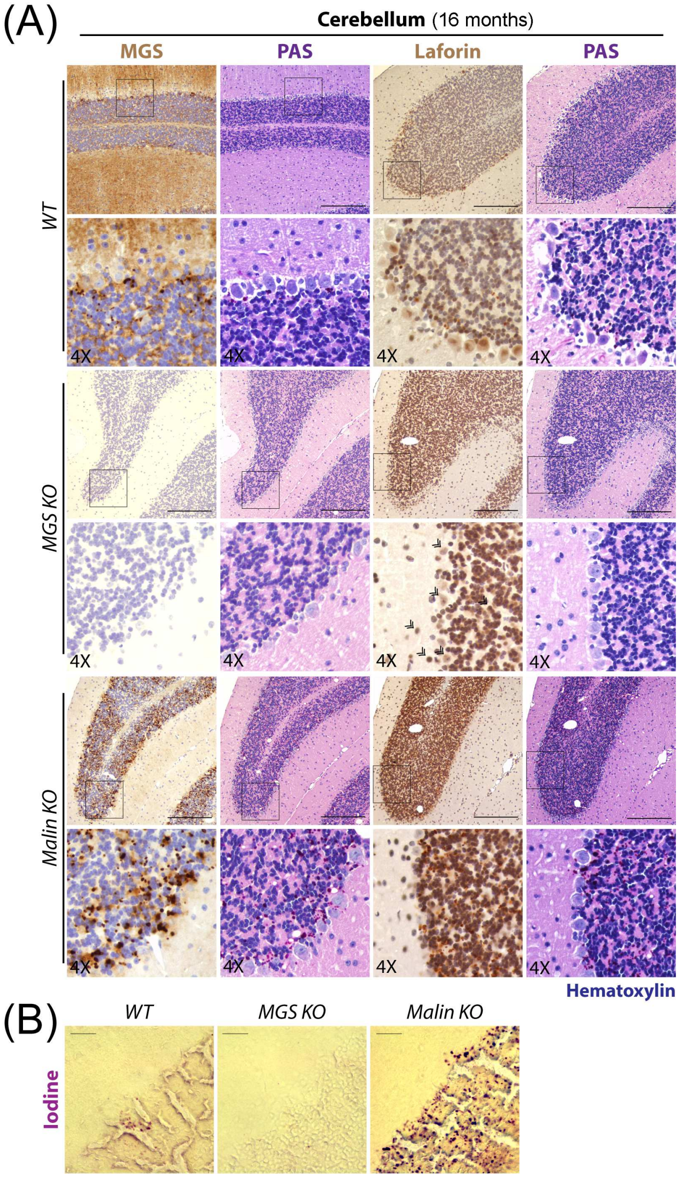

Figure S3. MGS and laforin proteins accumulated with polyglucosan bodies (PGBs) in the cerebellum of aged WT and malin KO mice. Aged MGS KO mice did not show accumulations. Cerebella from 16-month-aged WT, malin KO and MGS KO mice are shown. (A) 4-µm-thick consecutive brain sections stained with periodic acid-Schiff (PAS) and immunostained for MGS and laforin (brown) as indicated. All the sections were counterstained

with hematoxylin (blue). Laforin stainning presented nuclear localization in MGS KO mice (arrowheads). Scale bar = 200µm, 4X = 4-fold magnification. (B) Iodine staining. Scale bar = 30µm.

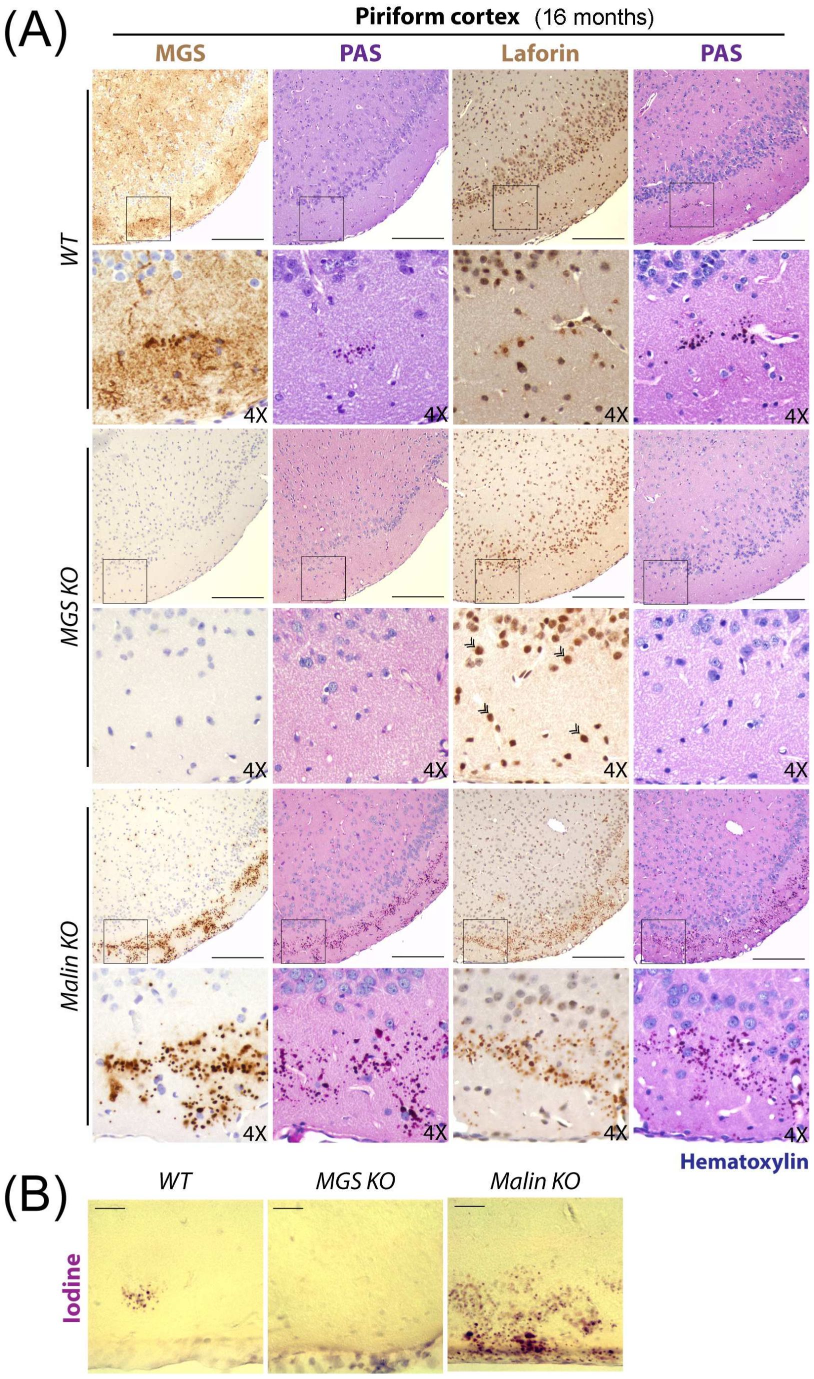

Figure S4. MGS and laforin proteins accumulated with polyglucosan bodies (PGBs) in the piriform cortex of aged WT and malin KO mice. Aged MGS KO mice did not show

accumulations. Piriform cortexes of 16- month-old WT, malin KO and MGS KO mice are shown. (A) 4-µm-thick consecutive brain sections stained with periodic acid-Schiff (PAS) and immunostained for MGS and laforin (brown) as indicated. All the sections were counterstained with hematoxylin (blue). Laforin stainning presented nuclear localization in MGS KO mice (arrowheads). Scale bar = 200µm, 4X = 4-fold magnification. (B) Iodine staining. Scale bar = 30µm.

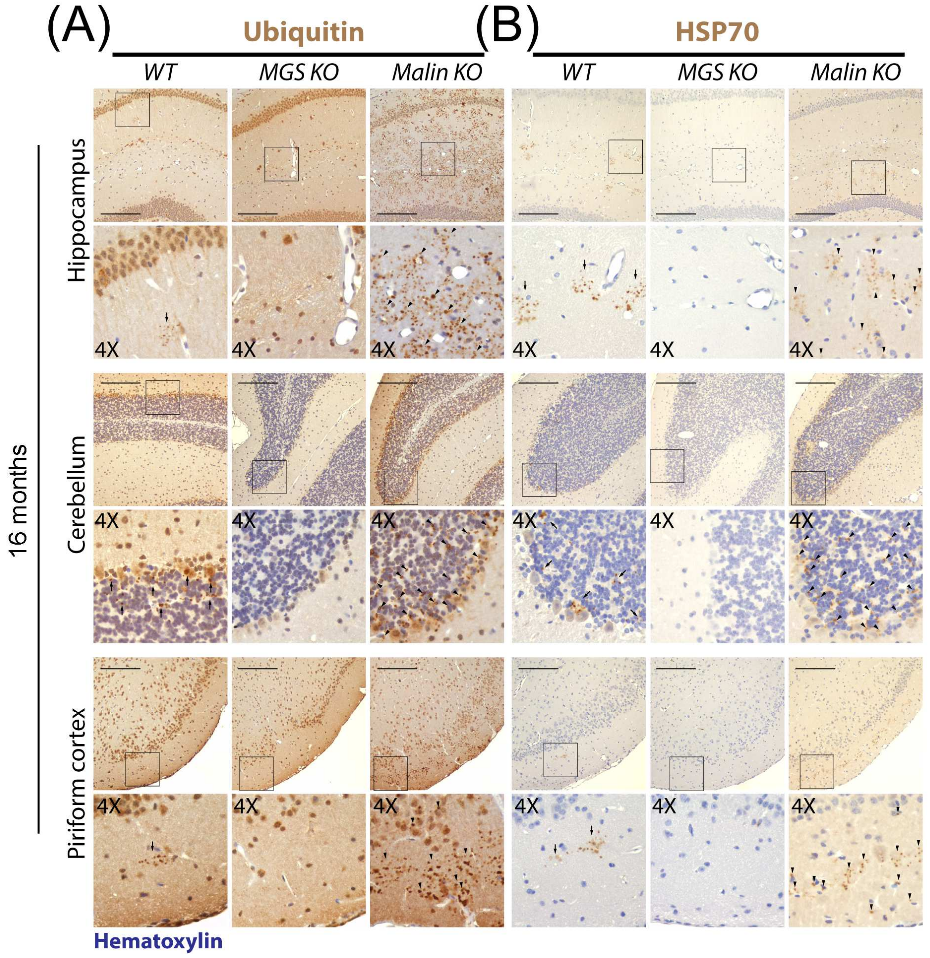

Figure S5. Ubiquitin and 70 kDa heat shock protein (HSP70) accumulated with polyglucosan bodies (PGBs) in aged WT and malin KO mice. Aged MGS KO mice did not show accumulations. Hippocampus, cerebellum and piriform cortex regions of 16-month-old WT, malin KO and MGS KO mice are shown. 4-µm-thick sections consecutive to those stained with periodic acid-Schiff (PAS) were immunostained (brown) with antibodies against ubiquitin

(A) and 70 kDa heat shock protein (HSP70, B). All the sections are counterstained with hematoxylin (blue). Ubiquitin and HSP70-positive PGBs were found in aged WT (arrows) and malin KO mice (arrowheads) Scale bar = 200µm, 4X = 4-fold magnification.

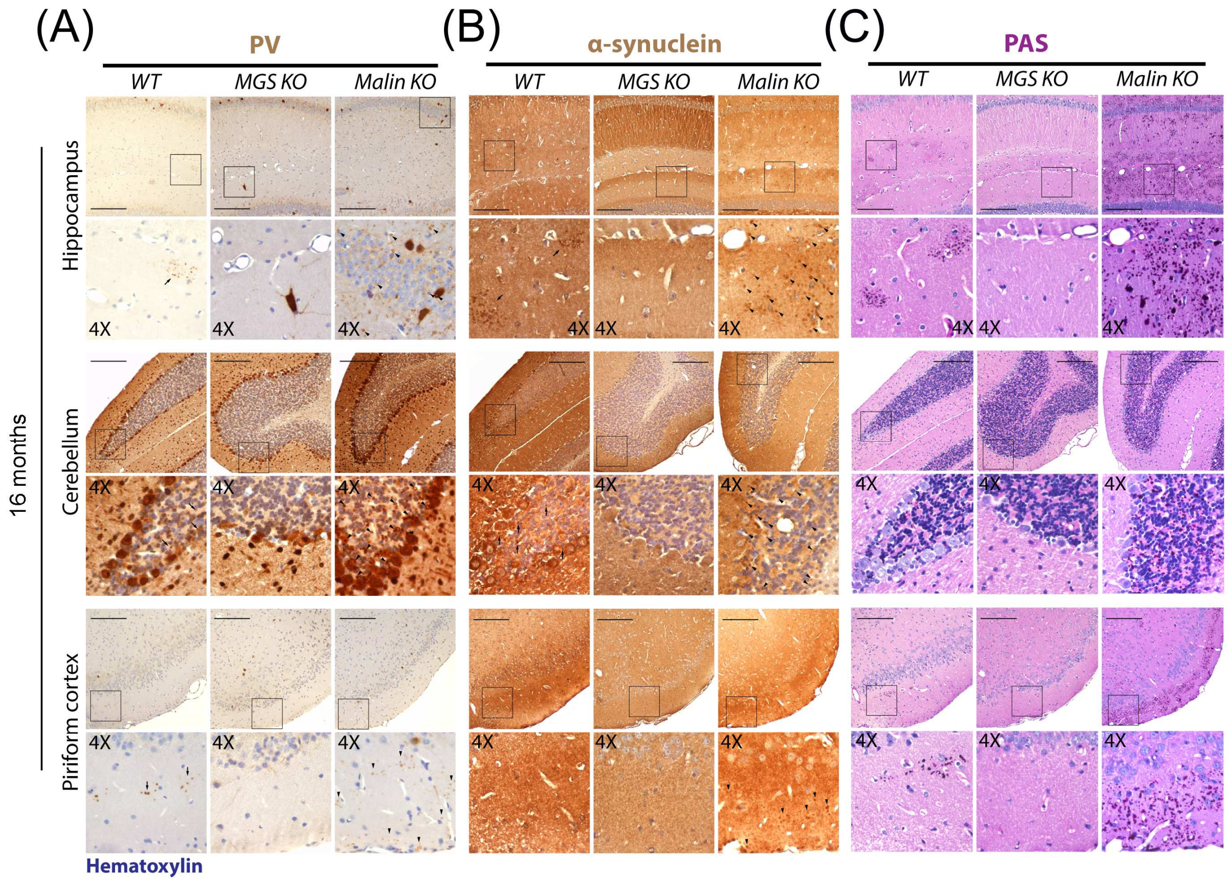

Figure S6. Parvalbumin (PV) and alpha-synuclein accumulated with polyglucosan bodies (PGBs) in aged WT and malin KO mice. Aged MGS KO mice did not show accumulations. Hippocampus, cerebellum and piriform cortex regions from 16-month-old WT, malin KO and MGS KO mice are shown. 4-µm-thick sections consecutive to those stained with periodic acid-Schiff (PAS, C) were immunostained (brown) with antibodies against parvalbumin (PV, A) and alpha-synuclein (B). All the sections are counterstained with hematoxylin (blue). PV and alpha-synuclein were found in aged WT (arrows) and malin KO mice (arrowheads) Scale bar = 200µm, 4X = 4-fold magnification.

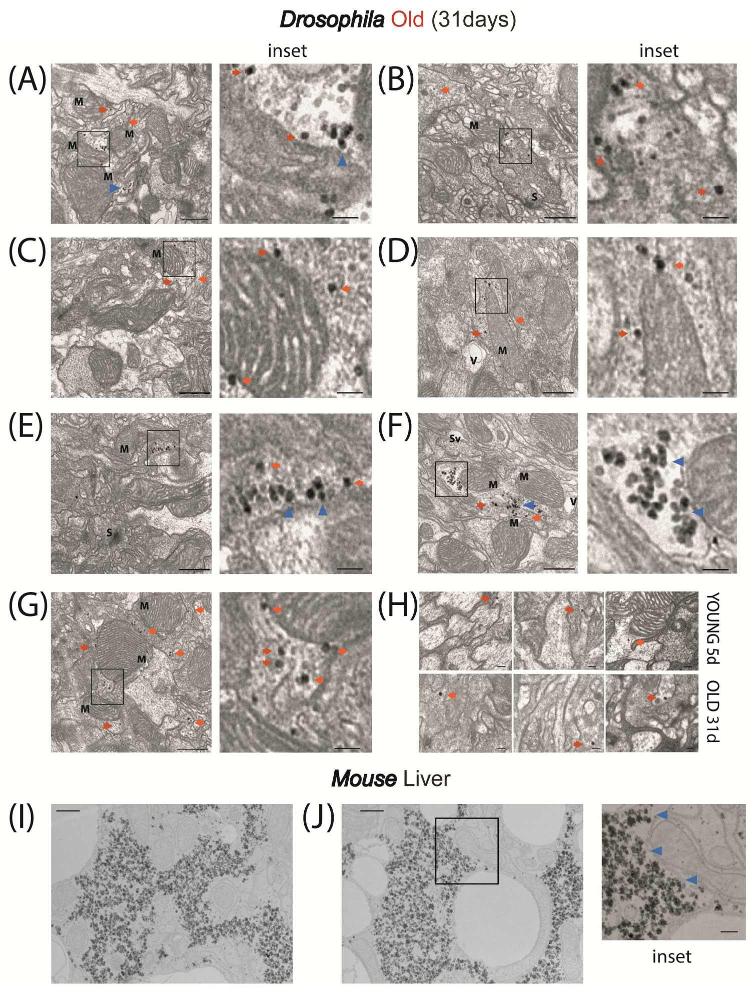

Figure S7. Glycogen accumulation in aged *Drosophila* as compared to mouse liver glycogen. A-G, Transmission electron microscopy (TEM) images (x26500) of optic lobe neuropil from *Drosophila* (*e*> *w*^{1118}) aged at 29°C to 31d. Arrow (red) – glycogen granule, arrow head (blue) – glycogen cluster, M – mitochondrion near to glycogen, V – vacuole, Sv – process containing synaptic vesicles. Scale bar 500 nm, inset 100 nm. (H) Comparison of the largest glycogen granules selected from TEM images of optic lobe neuropil in young (5d) or old (31d) animals. Scale bar 100nm. (I, J) Example images of mouse liver glycogen observed using the Thiéry method. Arrow (red) – glycogen granule, arrow head (blue) – glycogen cluster/rosette. Scale bar 500nm, inset 100nm.

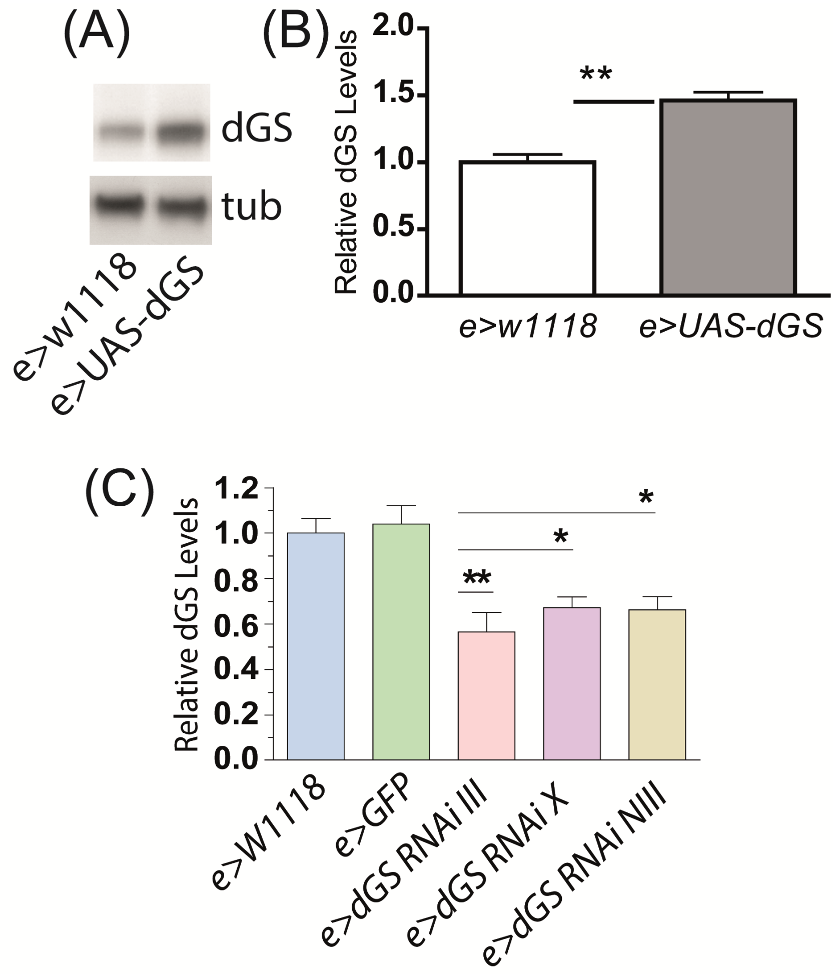

Figure S8. **Modulation of neuronal dGS levels detected in whole fly heads using anti-human MGS antibody.** (A) Representative Western blot from whole head homogenates of female flies (5d) overexpressing UAS-dGS construct driven by elav-GAL4 in neurons (e>). (B) Quantification of average dGS band density corrected to tubulin control for panel A, n=3 experiments. (C) Quantification of average dGS band density corrected to tubulin control for figure 4D, n=5 experiments. *p<0.05, ** p<0.01. Western blot membranes were exposed to anti-hMGS antibody (3886 clone, Cell Signaling, 1/1000). tub – tubulin loading control.

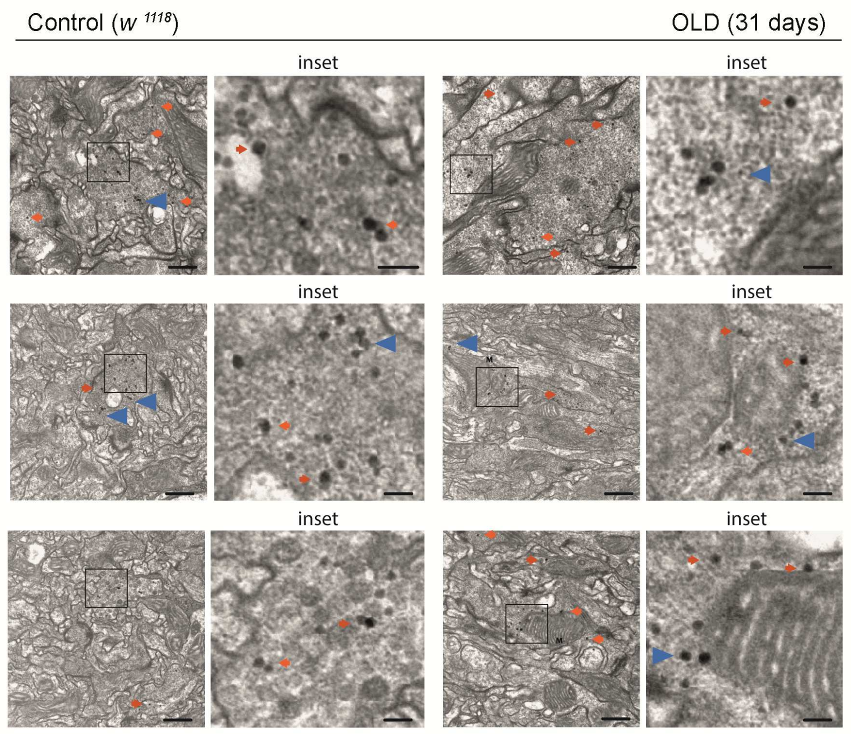

Figure S9. Identification of neuronal glycogen and glycogen clusters in aged *w*^{1118} strain flies. TEM images (x26500) of optic lobe neuropil from flies that were aged for 31d at 29°C. Arrow (red) –neuronal glycogen granule, arrow head (blue) – neuronal glycogen cluster, M – mitochondrion near to glycogen. Scale bar 500 nm, inset 125 nm.

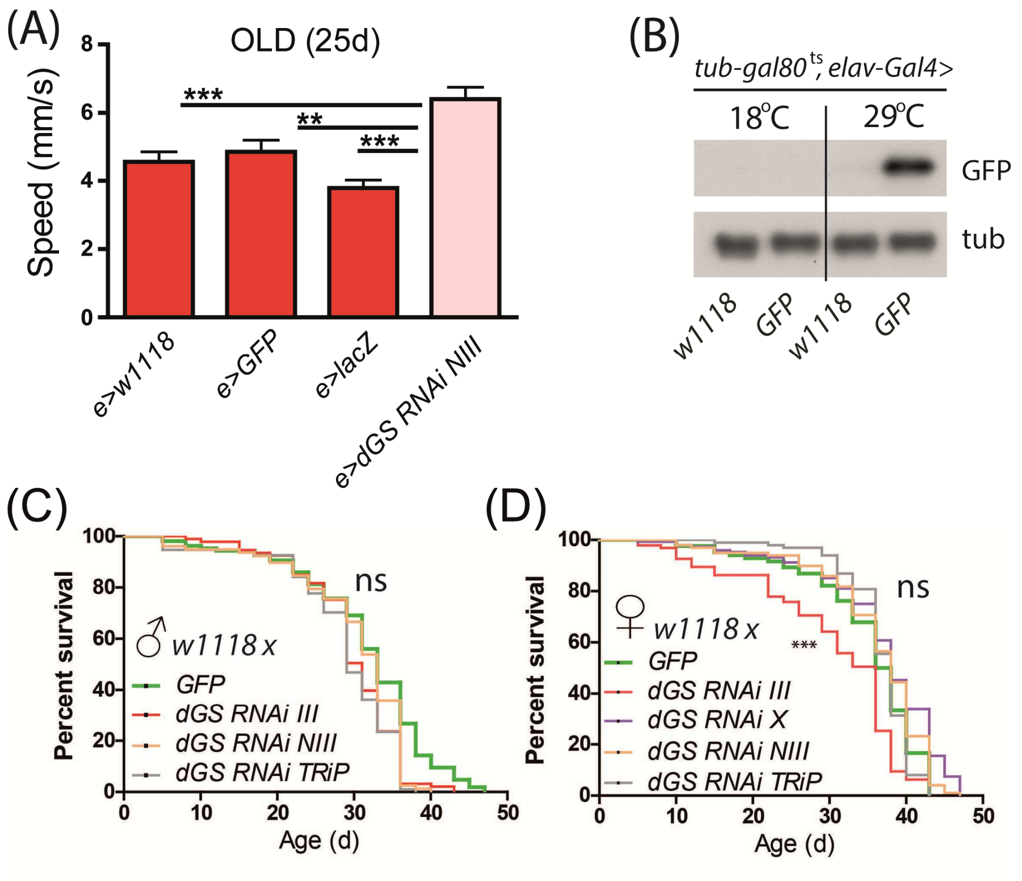

Figure S10. Data supporting neuronal RNAi knockdown of dGS and its functional consequences. (A) Quantification of climbing speed following vial tap for aged (25d) neuronal dGS RNAi flies (pink) versus several standard genetic control genotypes (dark red). (B) Gal80^{ts}-mediated temperature-sensitive conditional expression of UAS-GFP construct. GFP is detected only at the permissive temperature (29°C), demonstrating the efficacy of the system for adult-specific dGS RNAi in neurons. tub – tubulin. (C, D) UAS parental line control lifespan curves for males (C) and females (D). Median lifespans and number of animals tested for comparisons of interest: C, GFP 33d, n=106; dGS RNAi III 31d, n=93, dGS RNAi NIII 33d, n=93, dGS RNAi TRiP 29d, n=94; D, GFP 37d, n=84; dGS RNAi III 36d, n=95; dGS RNAi X 38d, n=148; dGS RNAi NIII 38d, n=99, dGS RNAi TRiP 38d, n=99. *** p<0.001 for reduction in lifespan.

Movie M1. Climbing of *elav-Gal4>GFP* young flies. 9 day old flies crawling performance following vial tap as described in supplementary methods. Video is shown in real time. Scale bar is 1cm.

Movie M2. Climbing of *elav-Gal4>dGS RNAi NIII* young flies. 9 day old flies crawling performance following vial tap as described in supplementary methods. Video is shown in real time. Scale bar is 1cm.

Movie M3. Climbing of *elav-Gal4>GFP* aged flies. 25 day old flies crawling performance following vial tap as described in supplementary methods. Video is shown in real time. Scale bar is 1cm.

Movie M4. Climbing of *elav-Gal4>dGS RNAi NIII* aged flies. 25 day old flies crawling performance following vial tap as described in supplementary methods. Video is shown in real time. Scale bar is 1cm.

Table S1: Descriptive statistics for *Drosophila* lifespans

RNAi Line	Driver Cell type	Median Lifespan (Log Rank)		Maximum Lifespan (Wang-Alison)	
		Male	Female	Male	Female
dGS RNAi III	*elav-Gal4c* neurons	**** p<0.0001 n=120,119	** p=0.005† n=120,120	**** p<0.0001 n=120,120	ns n=120,120
dGS RNAi X	*elav-Gal4c* neurons	____________	ns n=120,120	____________	ns n=120,120
dGS RNAi NIII	*elav-Gal4c* neurons	**** p<0.0001 n=120,118	ns n=120,120	**** p<0.0001 n=120,120	ns n=120,120
dGS RNAi III	*None UAS only*	ns n=106,93	**** p<0.0001† n=84,95	ns n=106,93	* p=0.05† n=84,95
dGS RNAi X	*None UAS only*	____________	ns n=84,148	____________	ns n=84,148
dGS RNAi NIII	*None UAS only*	ns n=106,78	ns n=84,99	ns n=106,78	ns n=84,99
dGS RNAi TRiP	*None UAS only*	ns n=106,94	ns n=84,99	ns n=106,94	ns n=84,99

Statistical comparison is for each RNAi line versus controls. n values are listed as n=control,RNAi line. Significance values are for lifespan extension (except where otherwise stated) vs the *w1118*/*elav-Gal4c* (green) or GFP/w1118 (lilac) control. *elav-Gal4c* refers to neuron specific *elav-Gal4* line combined with $Gal80^{ts}$ (conditional expression adulthood only). UAS only lines (lilac) were derived from crossing all lines to *w1118* strain.
**** p<0.0001, ** p<0.01, * p<0.05, ns – not significant
† Significance for shortening of lifespan.
